# Novel *KIAA0753* mutations extend the phenotype of skeletal ciliopathies

**DOI:** 10.1038/s41598-017-15442-1

**Published:** 2017-11-14

**Authors:** A. Hammarsjö, Z. Wang, R. Vaz, F. Taylan, M. Sedghi, K. M. Girisha, D. Chitayat, K. Neethukrishna, P. Shannon, R. Godoy, K. Gowrishankar, A. Lindstrand, J. Nasiri, M. Baktashian, P. T. Newton, L. Guo, W. Hofmeister, M. Pettersson, A. S. Chagin, G. Nishimura, L. Yan, N. Matsumoto, A. Nordgren, N. Miyake, G. Grigelioniene, S. Ikegawa

**Affiliations:** 1 Department of Molecular Medicine and Surgery, Center for Molecular Medicine, Karolinska Institutet, Stockholm, Sweden; 2 Clinical Genetics, Karolinska University Hospital, Stockholm, Sweden; 3 Laboratory for Bone and Joint Diseases, RIKEN Center for Integrative Medical Sciences, Tokyo, Japan; 4 Department of Medical Genetics, Institute of Basic Medical Sciences, Peking Union Medical College, Chinese Academy of Medical Sciences, Beijing, China; 5 Medical Genetics Laboratory, Alzahra University Hospital, Isfahan University of Medical Sciences, Isfahan, Iran; 6 Department of Medical Genetics, Kasturba Medical College, Manipal University, Manipal, India; 7 The Prenatal Diagnosis and Medical Genetics Program, Department of Obstetrics and Gynecology, Mount Sinai Hospital, University of Toronto, Toronto, Ontario Canada; 8 Division of Clinical and Metabolic Genetics, Department of Pediatrics, The Hospital for Sick Children, University of Toronto, Toronto, Ontario Canada; 9 Department of Pathology and Laboratory Medicine, Mount Sinai Hospital, University of Toronto, Toronto, Ontario Canada; 10 Medical Genetics, Kanchi Kamakoti Childs Trust Hospital, Chennai Tamilnadu, India; 11 Department of Pediatric Neurology, Faculty of Medicine, Child Growth and Development Research Center, Isfahan University of Medical sciences, Isfahan, Iran; 12 Student Research Committee, Department of Modern Sciences and Technologies, Faculty of medicine, Mashhad University of Medical Sciences, Mashhad, Iran; 13 Department of Physiology and Pharmacology, Karolinska Institutet, Stockholm, Sweden; 14 Intractable Disease Center, Saitama University Hospital, Saitama, Japan; 15 Department of Neurology, China-Japan Friendship Hospital, Beijing, China; 16 Department of Human Genetics, Yokohama City University Graduate School of Medicine, Yokohama, Japan

## Abstract

The skeletal ciliopathies are a heterogeneous group of disorders with a significant clinical and genetic variability and the main clinical features are thoracic hypoplasia and short tubular bones. To date, 25 genes have been identified in association with skeletal ciliopathies. Mutations in the *KIAA0753* gene have recently been associated with Joubert syndrome (JBTS) and orofaciodigital (OFD) syndrome. We report biallelic pathogenic variants in *KIAA0753* in four patients with short-rib type skeletal dysplasia. The manifestations in our patients are variable and ranging from fetal lethal to viable and moderate skeletal dysplasia with narrow thorax and abnormal metaphyses. We demonstrate that KIAA0753 is expressed in normal fetal human growth plate and show that the affected fetus, with a compound heterozygous frameshift and a nonsense mutation in *KIAA0753*, has an abnormal proliferative zone and a broad hypertrophic zone. The importance of *KIAA0753* for normal skeletal development is further confirmed by our findings that zebrafish embryos homozygous for a nonsense mutation in *kiaa0753* display altered cartilage patterning.

## Introduction

Ciliopathies, genetic disorders associated with ciliary dysfunction, have a wide variety of clinical features and affect nearly every organ, including kidneys, liver, retina, brain and bone, among others^1,2^. Skeletal ciliopathies are a rare group of diseases, currently divided into nine subtypes with significant genetic heterogeneity and phenotypic overlap^3^. The main features of skeletal ciliopathies include thoracic hypoplasia and shortening of the tubular bones, often with metaphyseal dysplasia and trident pelvis. Variable extraskeletal manifestations include structural and functional abnormalities of the brain, retina, genitalia, kidney, and liver^4^. So far, mutations in 25 genes, coding for cilia or basal body structural proteins and intraflagellar transport proteins, have been identified in association with skeletal ciliopathies and the number of genes causing these conditions keeps growing^2,4–9^.

In 2010, Lehman *et al*. observed overlapping features between non-skeletal ciliopathies such as Joubert syndrome (JBTS [MIM:213300]) and short-rib thoracic dysplasia (SRTD [MIM:208500]) in three families. It was suggested that the cause is a mutated ciliary gene coding for a protein important for both skeletal and cerebellar development^10^. KIAA0753 protein has been identified as a centrosome component involved in centriole duplication and interacting with other centrosomal proteins^11–13^. Pathogenic variants in *KIAA0753* have recently been found in association with JBTS^14^ and orofaciodigital syndrome (OFD15 [MIM:617127])^12^.

In this study, we report four patients with skeletal dysplasia and features of JBTS having novel deleterious variants in *KIAA0753*. They present with variability in the severity of the skeletal abnormalities ranging from prenatal lethality in one fetus to viability with moderate skeletal dysplasia in three children. In addition two of our patients showed brain abnormalities consistent with JBTS. Examination of the distal femoral growth plate of the affected fetus showed an abnormal proliferative zone. Zebrafish knockout model with homozygous nonsense variants in the *kiaa0753* gene show classic ciliopathy phenotype with cartilage abnormalities and curved body seen in other ciliopathy models^15^ and abnormal head morphology due to altered cartilage patterning and lethality at early developmental stages. These findings resemble our patients’ pathology and demonstrate the importance of *KIAA0753* in skeletal as well as brain development.

## Results

### Patients’ Reports

#### Patient 1

**Figure 1:**
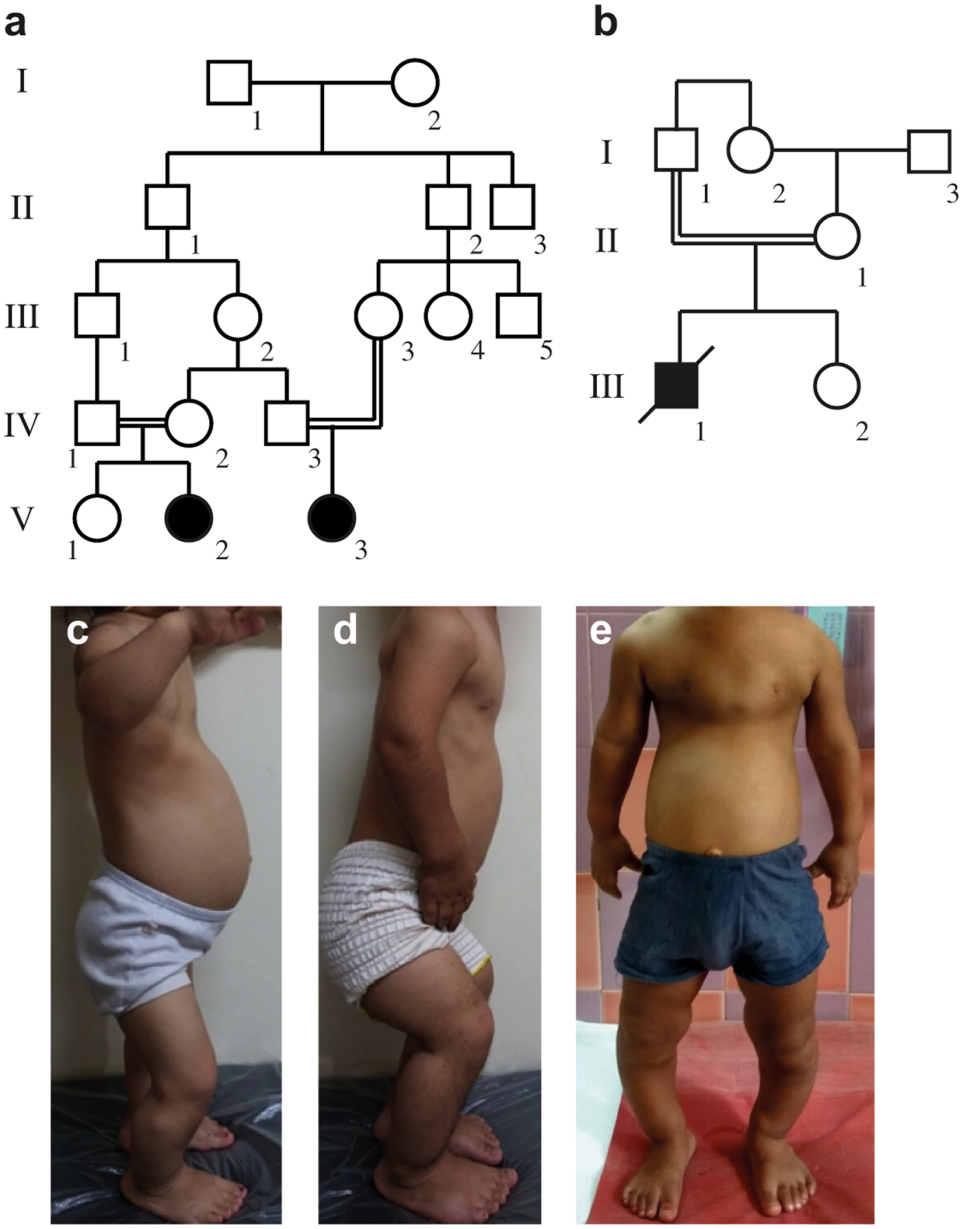
Pedigrees and clinical pictures of patients 1–3. (**a**) Pedigree of family 1 and (**b**) family 2 indicate consanguinity in both families, the patients are filled symbols (in a, P1 is V:3; P2 is V:2, in b P3 is III:1). (**c**) P1 at the age of 5 years and 10 months; (**d**) P2 at the age of 5 years and 9 months and (**e**) P3 at 6 years. Note narrow thorax in all patients, prominent abdomen in P1 and P3 and rhizomelic shortening of the upper limbs in P2 and P3. P2 has severe flexion contractures of the elbows, hips and knees, as well as genu varum.

**Figure 2:**
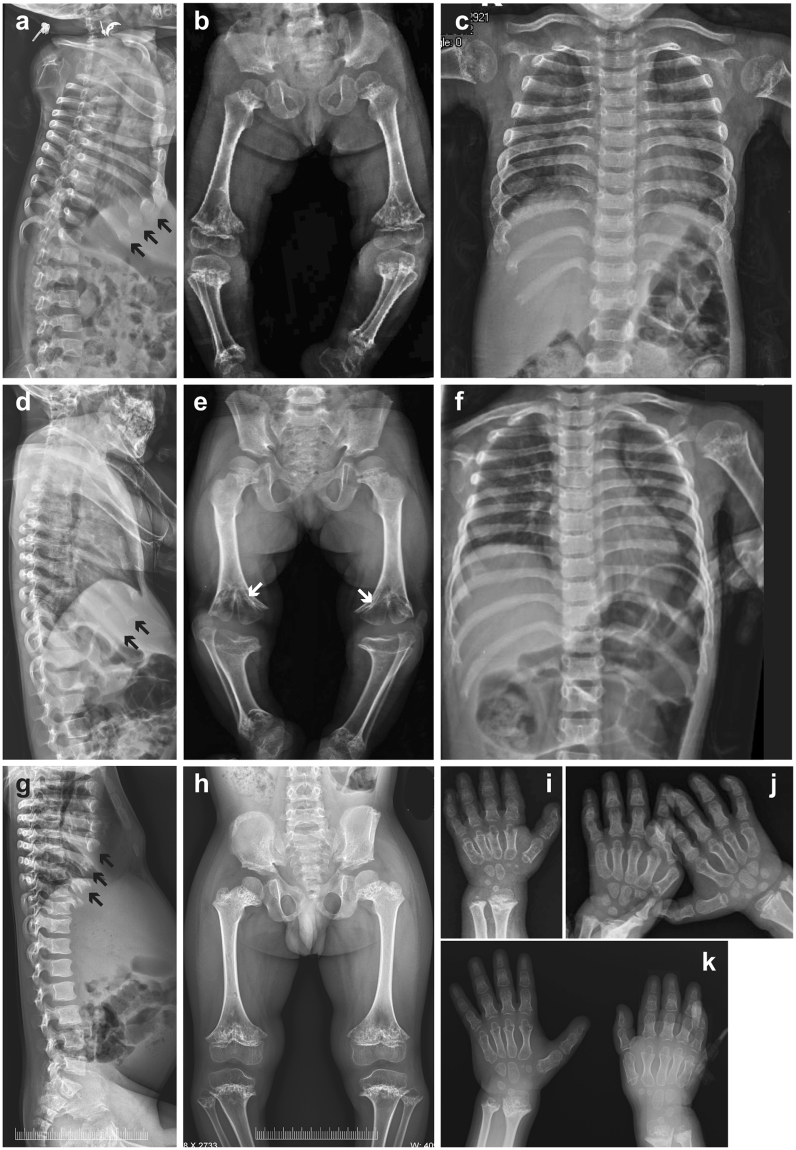
Skeletal radiograms of patients 1–3 (P1-P3). (**a**–**c**,**i**) P1 at age of 5 years and 10 months; (**d**–**f**,**j**) P2 at age of 5 years and 9 months; (**g**,**h**,**k**) P3 at the age of 6 years and 6 months. (**a**,**d**,**g**) Lateral spine. Note cupped anterior ends of the ribs (black arrows); (**b**,**e**,**h**) Pelvis and lower limbs. Short ilia, sclerotic iliac crest, narrow sciatic notch, horizontal acetabular roof, metaphyseal irregularities and sclerosis, metaphyseal flaring of the long tubular bones, short and curved tibia are seen; (**e**) Note schypodysplasia (white arrows) of the knees in P2 due to premature fusion of the growth plate; (**c**,**f**) Chest with short broad ribs, severe metaphyseal irregularity and sclerosis of the proximal humeri; (**i**–**k**) Hands. Note short phalanges and metacarpals. The distal radius and ulna show metaphyseal irregularities with irregular sclerosis.

Patient 1 (P1) is a 6-year-old girl born to a consanguineous healthy Iranian couple (V:3; Fig. 1a), following an uncomplicated pregnancy with an elective Cesarean section delivery at 39 weeks gestation. The birth measurements were normal (Supplementary Table S1). Generalized tonic-clonic seizures were noted in the newborn period and treated with Clonazepam. At 20 months of age, she had disproportional short stature with severe genu varum, relatively large head, depressed nasal bridge and low set ears. Her thorax was narrow and abdomen was slightly prominent, but no hepatosplenomegaly was found. She had delayed motor development (sitting unaided at 1 year; walking at 3 years of age). On follow-up at 6 years she had teeth hypoplasia, pectus excavatum, rhizomelic shortening of the limbs and flexion contractures of the elbows, knee and hips (Fig. 1c). Her vision and hearing were normal. Skeletal survey showed severe metaphyseal dysplasia and cone-shaped epiphyses of the proximal tibia and distal femur embedded in cup-shaped metaphyses (Fig. 2a–c). Phalangeal and metacarpal epiphyses were also cone-shaped and the metacarpals and phalanges were short (Fig. 2i). The spine and skull were unremarkable. Neurology examination showed moderate mental retardation and hypotonia. Blood samples including evaluation of the endocrine, liver and renal functions and urinary mucopolysaccharides were all normal, as well as abdominal ultrasound and echocardiography. Brain magnetic resonance imaging (MRI) showed hypoplasia of the corpus callosum, small pituitary gland, inferior vermis dysplasia, “molar tooth” sign (MTS) and mildly dilated third ventricle (Supplementary Fig. S1).

#### Patient 2

Patient 2 (P2) is first cousin of P1 and the first child born to a healthy consanguineous Iranian couple (V:2; Fig. 1a). She was born by an elective Cesarean section following an uncomplicated pregnancy at 39 weeks gestation. Her birth measurements were normal (Supplementary Table S1). A few episodes of generalized tonic-clonic seizures in the newborn period were treated with phenobarbital. She had disproportionate short stature with genu varum at 24 months of age. Investigations including liver and renal functions were normal. At her last follow-up at 6 years of age, she had similar clinical and radiographic features to her cousin, including teeth hypoplasia, skeletal dysplasia and psychomotor and speech delays (Figs 1d and 2d–f,j, Table 1). Brain MRI showed hypoplasia of the corpus callosum, a small pituitary gland, inferior vermis hypoplasia, MTS and mildly dilated lateral ventricles (Supplementary Fig. S2).

**Table 1:** Clinical characteristics of present and previously reported patients with mutations in *KIAA0753*.

	Patient 1	Patient 2	Patient 3	Patient 4	Patient 1 (Chevrier *et al*. 2016)	Patient 1 (Stephen *et al*. 2017)	Patient 2 (Stephen *et al*. 2017)
Clinical diagnosis	SKD + JBTS	SKD + JBTS	SKD + JBTS?	SRTD (fetus)	OFD	JBTS	JBTS
Disproportionate short-limb short stature (HP:0008873)	+	+	+	+	NA	+*	+*
Thoracic hypoplasia (HP:0005257)	+	+	+	+	NA	NA	NA
Hands	Brachydactyly (HP:0001156)	Brachydactyly (HP:0001156)	Brachydactyly (HP:0001156)	Brachydactyly (HP:0001156)	Polydactyly (HP:0010442)	−	−
Protuberant abdomen (HP:0001538)	+	−	+	+	NA	+	NA
Flexion contracture (HP:0001371)	+	+	−	NA	NA	NA	NA
Delayed gross motor development (HP:0002194)	+	+	+	NA	NA	−	+ (borderline)
Delayed speech and language development (HP:0000750)	+	+	+	NA	NA	+	+
CNS anomalies	Vermis dysplasia (HP:0002334), MTS (HP:0002419), corpus callosum hypoplasia (HP:0007370), dilation of lateral ventricles (HP:0006956), small pituitary gland (HP:0012506)	Vermis dysplasia (HP:0002334), MTS (HP:0002419), corpus callosum hypoplasia (HP:0007370), dilation of lateral ventricles (HP:0006956), small pituitary gland (HP:0012506)	Computer tomography of head was unremarkable, MRI not performed	Ventriculomegaly (HP:0002119), vermis dysplasia (HP:0002334)	Vermis hypoplasia (HP:0001320), MTS (HP:0002419), corpus callosum aplasia (HP:0007370), dilation of lateral ventricles (HP:0006956)	Vermis dysplasia (HP:0002334) and hypoplasia (HP:0001320), MTS (HP:0002419), ectopic posterior pituitary (HP:0011755)	Vermis dysplasia (HP:0002334) and hypoplasia (HP:0001320), MTS (HP:0002419), small pituitary gland (HP:0012506)
Craniofacial anomalies	Frontal bossing (HP:0002007), flat face (HP:0012368), depressed nasal bridge (HP:0005280), teeth hypoplasia (HP:0000685)	Frontal bossing (HP:0002007), flat face (HP:0012368), depressed nasal bridge (HP:0005280), teeth hypoplasia (HP:0000685)	Frontal bossing (HP:0002007), flat face (HP:0012368), low set ears (HP:0000369)	Wide nasal bridge (HP:0000431), low set, posteriorly rotated ears (right) (HP:0000368), short lingual frenulum (HP:0000200)	Flat face (HP:0012368), hypertelorism (HP:0000316), wide nasal bridge (HP:0000431), low set, posteriorly rotated ears (left) (HP:0000368), lobulated tongue (HP:0000180)	Flat face (HP:0012368), low set, posteriorly rotated ears (HP:0000368), oculomotor apraxia (HP:0000657)	Frontal bossing (HP:0002007), flat face (HP:0012368), low set, posteriorly rotated ears (HP:0000368), oculomotor apraxia (HP:0000657)
Growth hormone deficiency (HP:0000824)	Lower limit	−	NA	NA	NA	+ *	+*

SKD, skeletal dysplasia; JBTS, Joubert syndrome; SRTD, short-rib thoracic dysplasia; OFD, orofaciodigital syndrome; +, present; −, absent; NA, not available; MTS, molar tooth sign; MRI, magnetic resonance imaging; *, children responded to growth hormone therapy.

#### Patient 3

Patient 3 (P3) is the first offspring born to a consanguineous couple of Indian descent. He has a healthy younger sister (Fig. 1b). He was born at term following a normal pregnancy and delivery with normal birth weight and was seen initially in the clinic at the age of 6 years and 3 months for respiratory difficulties due to bilateral pneumonia. He had disproportionate short stature (Supplementary Table S1), speech delay, pectus carinatum, narrow chest, protuberant abdomen and short limbs (Figs 1e and 2g,h,k, Table 1). The skeletal phenotype resembled that of P1 and P2. Echocardiography revealed pulmonary hypertension. His hearing, ocular fundi and vision were normal. Abdominal ultrasonography showed mild hepatomegaly. Brain computed tomography scan was unremarkable. His complete blood counts, blood, biochemical studies and urinalysis were unremarkable. He succumbed due to respiratory failure following a respiratory infection, at the age of seven years.

#### Patient 4

**Figure 3:**
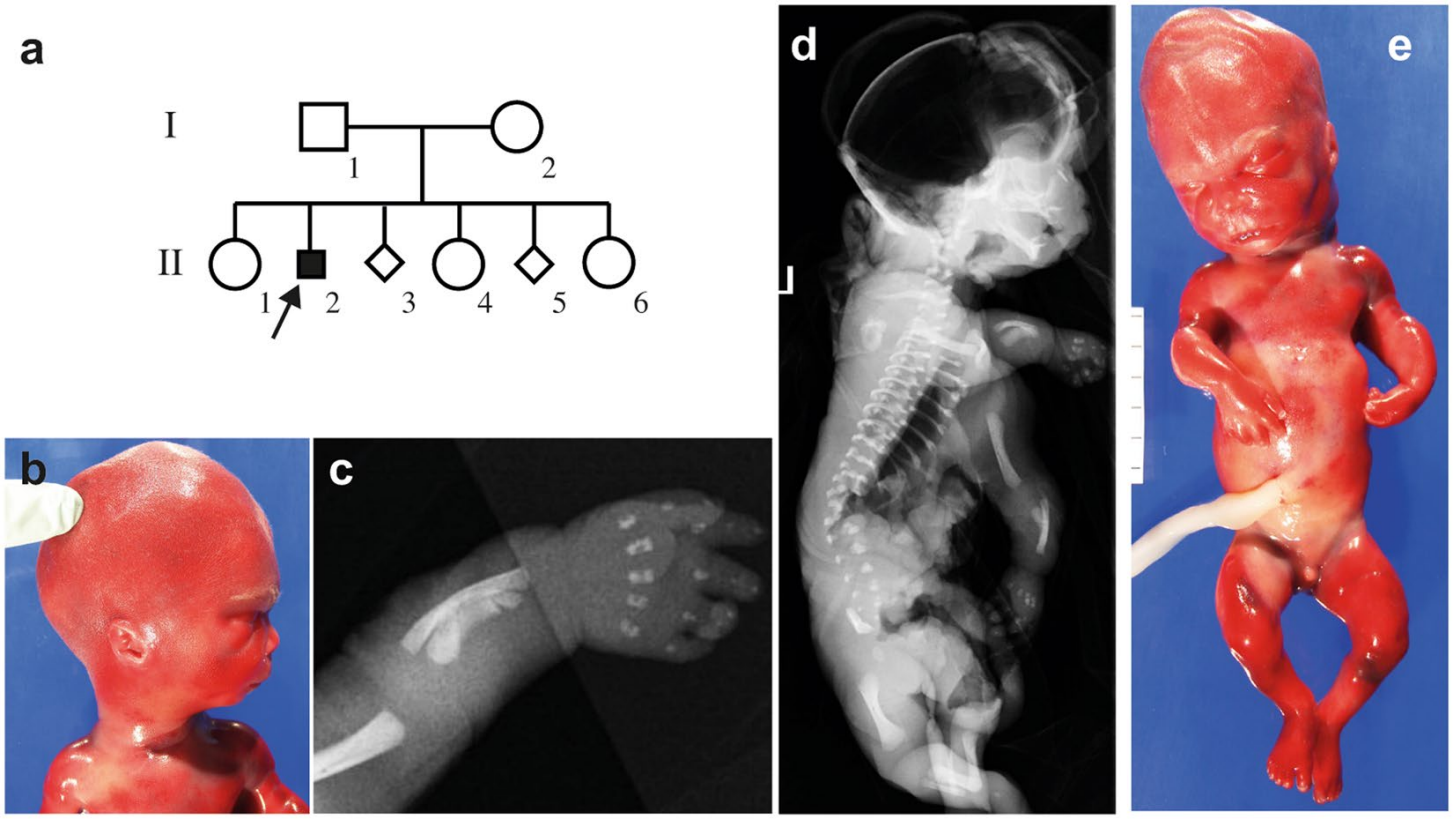
Radiograms, clinical photos and pedigree of patient 4 (P4). (**a**) The pedigree of family 3, P4 is from the second pregnancy (II-2) which was terminated at gestational age 19 + 3. Pregnancy number 3 (II-3) was a missed abortion in week 8 and pregnancy 5 of the same couple (II-5) was terminated at GA 25 due to similar features; (**b**,**e**) The clinical pictures showing distinctive facial features with a relatively large head, micrognathia, high forehead, low-set posteriorly rotated left ear and small nose with a broad nasal root. The abdomen is prominent and the external genitalia show a micropenis; (**c**,**d**) Radiograms of P4 show short bowed long bones, short ribs and extremely narrow thorax.

The parents of the affected fetus (P4) were healthy, non-consanguineous and of Italian origin (Fig. 3a). Fetal ultrasound at 19 weeks gestation showed short limbs, narrow chest, prominent abdomen, cerebral ventriculomegaly, suspected defect in the cerebellar vermis and mild pelviectasis. The parents chose to terminate the pregnancy and the fetal autopsy showed macrocephaly, wide open anterior and posterior fontanels, high forehead with frontal bossing, micrognathia, low-set and posteriorly rotated left ear, small nose with a broad nasal bridge, long philtrum, normal lips and palate, short lingual frenulum, short and bowed limbs, brachydactyly, micropenis and hypospadia (Fig. 3b,e). There were no internal malformations and the brain autopsy showed a relatively large brain (corresponding to 21-22 GA) and presence of germinal matrix excrescences. The eye histopathology was normal. Fetal radiography showed narrow thorax, trident ilia with spikes at the sacrosciatic notches and short bowed humeri, femora, tibiae, radii and ulnae (Fig. 3c,d). The fetal karyotype was normal male (46,XY) and array-CGH was normal. The findings on autopsy and radiography are summarized in Table 1 and Supplementary Tables S1 and S2.

#### Mutation analysis

Exome sequencing was performed on all patients (statistics of exome sequencing summarized in Supplementary Table S3). Pathogenic variants in *KIAA0753* (NM_014804.2) were found in all four affected individuals. In family 1 and 2, all three affected patients were homozygous for a nonsense mutation, c.970 C > T (p.Arg324*); and in family 3, the affected fetus was compound heterozygous for a nonsense variant c.943 C > T (p.Gln315*) and a 1-bp deletion, c.1271del (p.Pro424Hisfs*9). Sanger sequencing confirmed the variants, which segregated in an autosomal recessive mode of inheritance (Table 2 and Supplementary Fig. S3). The variants identified in our cohort are submitted in the ClinVar database (accession numbers SCV000580715, SCV000580733 and SCV000580734).

**Table 2:** Summary of *KIAA0753* variants in our and in previously reported patients.

Family	Individual	Nucleotide change^a^	Amino acid change	Comments
F1	P1 P2	c.970 C > T^b^	p.Arg324*	gnomAD, 4/245846 Het, MAF 1.627e-5 (South Asian and non-Finnish European)
F2	P3	c.970 C > T^b^	p.Arg324*	gnomAD, 4/245846 Het, MAF 1.627e-5 (South Asian and non-Finnish European)
F3	P4	c.943 C > T c.1271del	p.Gln315* p.Pro424Hisfs*9	gnomAD, 6/276744 Het, MAF 2.168e-5 (non-Finnish European) Not reported in gnomAD
OFD^12^	P1	c.1546-3 C > A c.1891A > T	p.Asp439Glyfs*5 p.Lys631*	gnomAD 2/246040 Het, MAF 8.129e-6 (non-Finnish European), rs886038200 gnomAD 2/246166 Het, MAF 8.125e-6 (non-Finnish European), rs886038201
JBTS^14^	P1 P2	c.769 A > G c.2359-1 G > C	p.Arg257Gly p.Lys787_Gln789del	Not reported in gnomAD Not reported in gnomAD

Het, heterozygous alleles; MAF, minor allele frequency; ^a^nucleotide change according to NM_014804.2 ^b^Homozygous for the variant.

Haplotype analysis shows that a founder event is unlikely between family 1 and 2, and that the variant in *KIAA0753* is a recurrent mutation (Supplementary Table S4). In P1 and P2 of family 1, a homozygous missense variant, c.425 C > T (p.Thr142Met), in *SLC13A5 (*NM_177550.4) was detected. This variant was not present in P3 or P4.

### Abnormal growth plate morphology and absent KIAA0753 expression in the affected fetus

**Figure 4:**
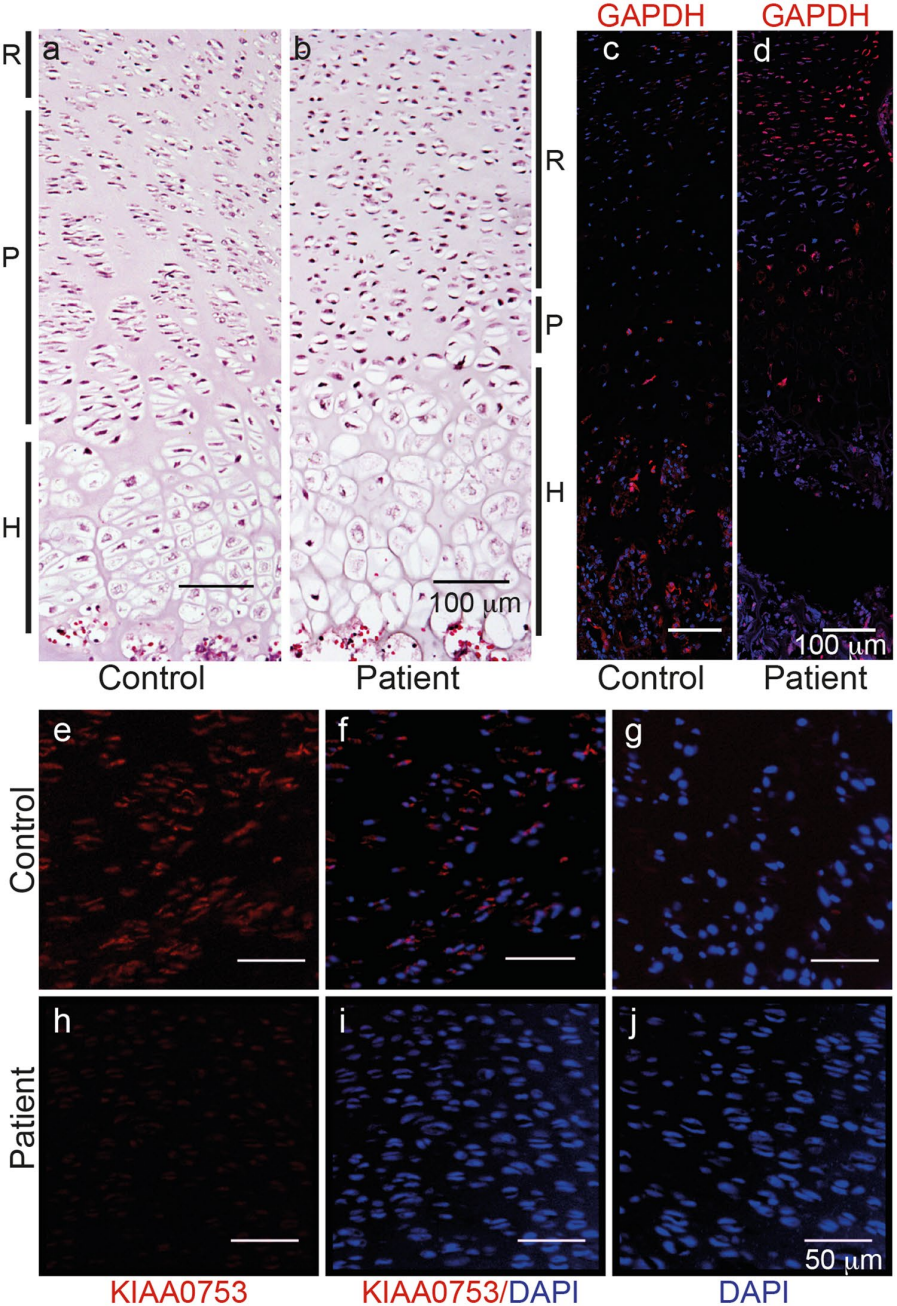
Histology and immunohistochemistry of the growth plate from normal control and affected fetus (P4). (**a**,**b**) Hematoxylin-eosin stained sections of the distal femoral growth plate from (**a**) normal control and (**b**) affected fetus, respectively. Zones of the growth plate are shown with black lines on the side. R, resting zone; P, proliferative zone and H, hypertrophic zone. Note the abnormal architecture of the proliferative zone in affected fetus with lack of normal chondrocyte columns; (**c**,**d**) GAPDH expression (red) is seen in the growth plate for the normal control and P4; (**e**–**j**) immunohistochemistry of the proliferative zone from paraffin embedded tissue. KIAA0753 expression (in red) is found in the normal control (**e**,**f**) but not in P4 (**h**,**i**); (**f**) and (**i**) the same area counterstained with DAPI for nuclear staining; (**g**,**j**) negative controls for the samples without primary antibody. Scale bar: 100 µm (**a**–**d**) and 50 µm(**e**–**j**).

Hematoxylin-eosin staining of the distal femoral growth plate from the control (Fig. 4a) and affected fetus (Fig. 4b) showed absence of normal columns in the proliferative zone and poorly organized broad hypertrophic zone in the affected fetus. Immunohistochemistry showed that KIAA0753 was expressed in the proliferative zone of normal control fetus (Fig. 4e,f), but absent in the corresponding cells from the affected fetus (Fig. 4h,i). Chondrocytes were also positive for KIAA0753 expression in the hypertrophic zone in the normal fetus, but the hypertrophic zone of the affected fetus detached during the antigen retrieval procedure repeatedly. GAPDH was used as a positive control for the immunohistochemistry for the normal fetus (Fig. 4c) and affected fetus (Fig. 4d).

### Expression of mutant *kiaa0753* in zebrafish results in abnormal head and body morphology

**Figure 5:**
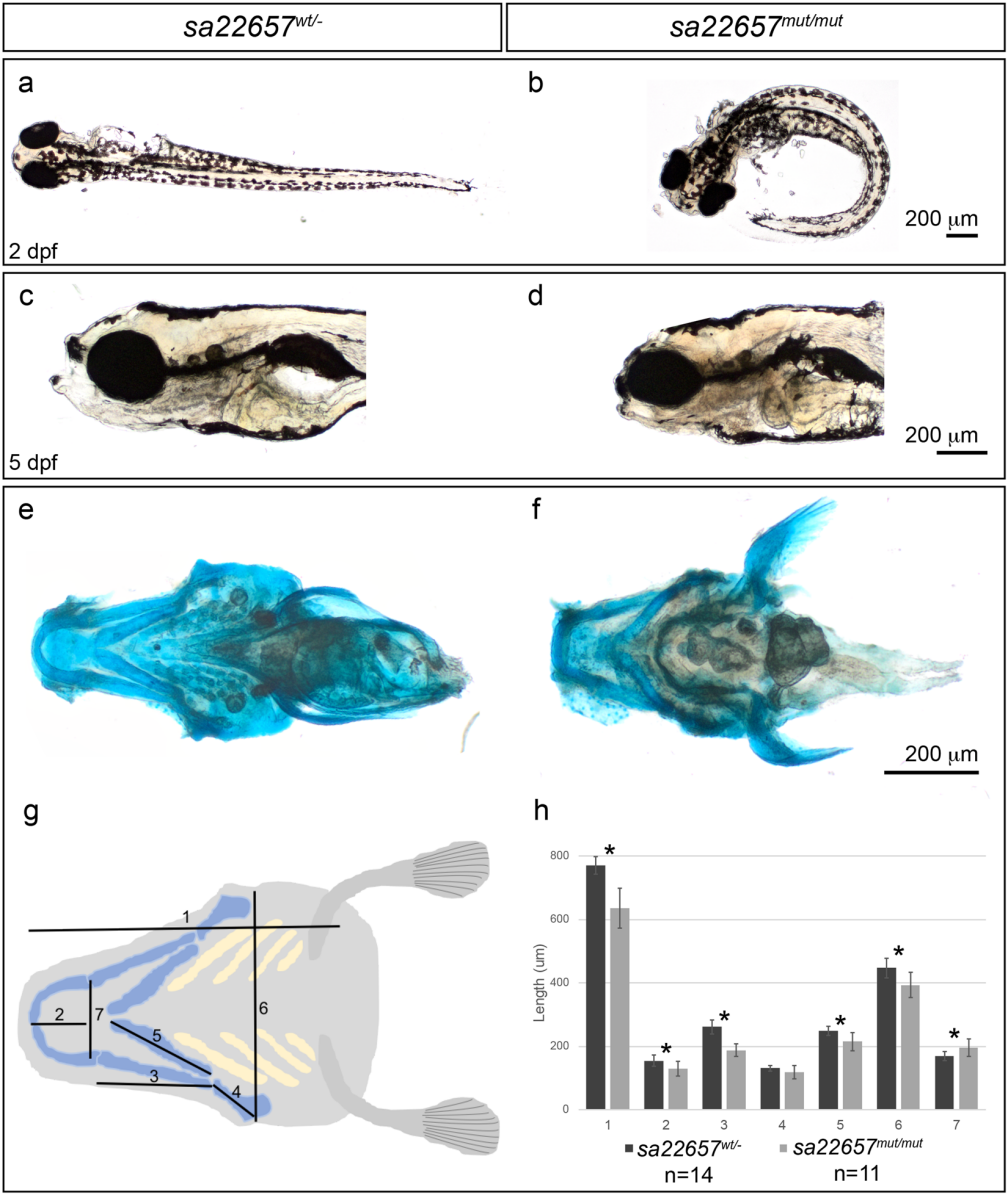
Skeletal morphogenesis is affected in zebrafish larvae expressing truncated *kiaa0753*. (**a**) Heterozygous and wild-type carriers have a straight body; (**b**) Zebrafish expressing mutant Kiaa0753 in homozygosity present a ciliopathy-like phenotype characterized by a curved body, visible from 2 dpf; (**c**) Normal larvae head at 5 dpf; (**d**) disruption of cartilage structures were evident by 5 dpf, with mutant larvae presenting shorter head length along the anterior-posterior axis; (**e**,**f**) To quantify the phenotype, alcian blue staining was performed in wild-type and mutants, respectively; (**g**), and cartilage patterning was quantified according to scheme; (**h**) Statistical analysis showed that measurements 1–3 and 5–7 showed a significant difference between wild-type and mutant larvae (*p < 0.01) (mutant, n = 11; wild-type, n = 14).

We used the zebrafish model to confirm the role of *kiaa0753* in ciliopathy and skeletal dysplasia. *In silico* protein sequence comparison showed that the human and zebrafish orthologues have 60.4% similarity. To evaluate the role of *kiaa0753* in skeletal morphogenesis, a commercially available mutant line (*sa22657* from EZRC) with a nonsense mutation resulting in truncated Kiaa0753 protein was used. Adult zebrafish were maintained in heterozygosity since homozygosity for the nonsense mutation in *kiaa0753* is lethal, with larvae not surviving beyond the first week. Genotype analysis of individual larvae showed that heterozygous larvae (*sa22657* ^*wt/mut*^) for the mutation in *kiaa0753* were phenotypically normal (Fig. 5a), while homozygous embryos (*sa22657* ^*mut/mut*^) presented with curved body as early as 2 days post fertilization (dpf) (Fig. 5b), a feature that is consistent with a cilia defect.

#### The *kiaa0753* mutants develop skeletal abnormalities

Since all four patients had skeletal dysplasia, we analyzed the cartilage structure in the developing zebrafish larvae and observed dysmorphology of the head of the homozygous mutant larvae (Fig. 5c,d). At 5 dpf, larvae were stained with a proteoglycan-staining dye, alcian blue. Overall, the structures of the skull were compressed in the anterior-posterior axis when compared to wild-type siblings (Fig. 5e,f). To quantify the differences, we compared the ventral head structure and measured the cartilage pattern in the zebrafish larvae head (7 measurements/larvae, according to scheme in Fig. 5g). We found that most cranial structures of the mutant larvae (n = 11) were significantly shorter than those of the wild-type siblings (n = 14, measurements 1–3, 5, and 6, p < 0.01), and that the most anterior part of the head was wider than wild-type larvae (measurement 7, Fig. 5h). These results support the involvement of KIAA0753 in the morphogenesis of the cranial bones.

## Discussion

We report, for the first time, that mutations in the *KIAA0753* gene cause skeletal dysplasia with narrow thorax and metaphyseal widening with limb bowing. In P4, the condition was lethal with skeletal changes including thorax hypoplasia and bowed limbs, while in the other three patients (P1-3), the phenotype included short stature, metaphyseal dysplasia with limb deformities, brachydactyly, developmental disability and features of Joubert syndrome.

Previously, biallelic deleterious variants in *KIAA0753* were reported in a patient with orofaciodigital syndrome (OFD) type 6 (OMIM currently classified as OFD15 [MIM:617127])^12^ and in two siblings with JBTS^14^. None of the clinical reports include skeletal dysplasia as a feature in their patients. The previously reported patients with JBTS were short (height at −3.3 and −3.7 z-scores, respectively, before growth hormone (GH) treatment), but no details were available regarding their skeletal features. These patients had GH deficiency and were successfully treated with GH^14^.

P1–3 in our study had developmental delay and brain abnormalities as in JBTS, which is consistent with previous report. The features of the affected fetus in our study resemble the facial dysmorphism reported by Chevrier *et al*. in the OFD patient (see Supplementary Table 1). Our patients with skeletal abnormalities seem to be the most severe end of *KIAA0753*-related congenital syndrome with combination of skeletal dysplasia and JBTS.

The differential diagnoses for the condition of our patients include other skeletal ciliopathies with metaphyseal involvement, such as: axial spondylometaphyseal dysplasia (SMDAX [MIM:602271]), Mainzer-Saldino syndrome (SRTD9 [MIM:266920]) and OFD syndrome type VI (OFD6 [MIM:258860]). The first two conditions are phenotypically excluded by lack of retinal degeneration and less conspicuous signs of metaphyseal dysplasia in the tubular bones. SRTD9 is also excluded because of lack of renal dysplasia and cerebellar ataxia in our patients. The broad metaphyses overlap with OFD6, but the absence of polydactyly and presence of mesomelic bowing of long tubular bones in our patients excludes this diagnosis. The cupped metaphyses and cone-shaped epiphyses of knees with brachydactyly in patient 1 and 2 raised the possibility of acroscyphodysplasia (MIM:250215); however, this condition has recently been found to be associated with mutations in the GNAS locus^16^ and our patients did not have disease causing variants in the *GNAS* gene, nor in the genes responsible for the other above mentioned differential diagnoses. Furthermore, our patients’ signs of short ribs and brain abnormalities are not typical for acroscyphodysplasia^17^.

Skeletal ciliopathies show a broad phenotype and genotype variability. Deleterious variants in genes coding for different ciliary components affect the function of the cilia in a similar way, leading to overlapping phenotypes and *vice versa*, mutations in the same gene may lead to a broad phenotypic spectrum^2,3^. For example, mutations in *CEP120* can lead to JBTS, OFD^18^ and severe short-rib thoracic dysplasia type 13 (SRTD13 [MIM:616300])^19^. Our patients with phenotypic features of skeletal dysplasia and brain abnormalities, together with previously reported patients with milder phenotypes of OFD and JBTS, represent the full phenotype-spectrum of the *KIAA0753*-related ciliopathy syndrome.

The newborn reported by Chevrier *et al*. with OFD was compound heterozygous for variants c.1891A > T (p.Lys631*) and splice mutation, c.1546-3 C > A (p.Asn439Glyfs4*) in *KIAA0753*. Both this newborn with OFD and our patients have premature stop codons occurring before the last splice junction, and therefore they are likely to lack KIAA0753 protein due to nonsense mediated decay^20^. EBV-immortalized B lymphocytes from the OFD patient showed weakly positive staining of KIAA0753 at the centrosomes, indicating that the splice mutation leads to residual production of small amounts of normal protein^12^. Residual KIAA0753 expression may explain milder skeletal abnormalities as in OFD syndrome, compared to more severe skeletal dysplasia features in our patients. Stephen *et al*. reported two siblings with JBTS who were compound heterozygous for a missense variant, c.769 A > G (p.Arg257Gly) and a cryptic splice site variant, c.2359-1 G > C, leading to in-frame deletion of three amino acids in KIAA0753. Fibroblasts from these patients had normal length of the cilia, but fewer ciliated cells^14^. The reason for these patients’ relatively mild phenotype is most probably because the variants also lead to some residual KIAA0753 expression. Considering the facts above, we hypothesize that the skeletal abnormalities detected in our patients are due to protein truncating variants, leading to absent KIAA0753, as confirmed by immunohistochemistry in the growth plate from the affected fetus.

In family 1, we also identified an additional homozygous missense variant, c.425 C > T (p.Thr142Met) in *SLC13A5* (NM_177550), a gene located ~44 kb upstream of *KIAA0753*. This mutation has been reported previously to cause autosomal recessive early infantile epileptic encephalopathy 25 (EIEE25 [MIM:615905]), a condition associated with seizures, developmental disability and teeth hypoplasia^21,22^. Extended SNP analysis from the exome data indicate that family 1 and 2 do not have a common ancestor and patient 3 did not have teeth hypoplasia or seizures. We thus conclude that the seizures and teeth hypoplasia in P1 and P2 are due to the homozygous *SLC13A5* variant, while the developmental and speech delay is an overlapping feature and could be inferable to either one of the congenital conditions. The findings of two rare conditions rather than one disease with phenotypic expansion affecting these two patients, is not an unusual situation in consanguineous families or isolated populations^23,24^.

In addition to the reports where *KIAA0753* was shown to code for a centrosome and pericentriolar satellite protein with important function in the signal transduction, we showed that it is expressed in the human fetal growth plate. Absence of functional KIAA0753 leads to growth plate abnormalities mainly in the proliferative and hypertrophic zones. The cilia maintain the organization of the growth plate, where the chondrocytes form columns in the proliferative zone^25^. The disorganized growth plate in mouse models with skeletal ciliopathy leads to abnormal growth because of lack of cell polarity and/or decreased rate of chondrocyte proliferation and differentiation^26,27^. Consistently, the growth plate structure of the fetus with *KIAA0753* mutations showed abnormalities in the proliferative and hypertrophic layers of the femoral growth plate. Our results indicate that the effect that the *KIAA0753* mutations have on cilia and centrosomes are sufficient to affect the growth plate organization, leading to severe postnatal short stature and bone deformities.

Zebrafish larvae with nonsense mutation in *kiaa0753* presented with a curved body, a well-known hallmark of cilia defects in zebrafish^15,28–33^ and disrupted morphogenesis of the cartilage of the head, which correlate with the skeletal phenotype in our patients.

In summary, we report severe skeletal dysplasia in four patients with deleterious variants in *KIAA0753* and demonstrate expression of KIAA0753 in the growth plate of a normal human fetus. We propose that *KIAA0753*-related syndrome spectrum is a phenotypic continuum from lethal skeletal dysplasia and JBTS features at the most severe end, through non-lethal metaphyseal skeletal dysplasia with short ribs and OFD to JBTS only at the mildest phenotypic end of the spectrum. Our study expands the phenotypic variability of the *KIAA0753*-related conditions and shows the importance of using massive parallel sequencing to achieve the diagnosis in view of the genetic and phenotypic heterogeneity of ciliopathies.

## Methods

### Patients

These patients were selected from studies on searching for molecular causes of ultra-rare skeletal dysplasias at Karolinska Institutet, Stockholm, Sweden, RIKEN Center for Integrative Medical Sciences, Tokyo, Japan and Kasturba Medical College, Manipal University, Manipal, India. The study was performed with ethical permissions from Karolinska Institutet (2014/983-31/1, 2013/1325-31/2), Isfahan University of Medical Sciences, Iran (2014/293058), RIKEN (Yokohama H16-40) and the Kasturba Hospital, Manipal (IEC:430/2013). The parents of the affected children and fetus provided written informed consent for the study. Evaluation of the families included family history, clinical examination including skeletal surveys and pathology report of the fetus from the terminated pregnancy. All methods were carried out in accordance with the relevant guidelines and regulations and the datasets generated during the current study are available from the corresponding authors on reasonable request.

### Exome sequencing and variant calling

Exome capture was performed on genomic DNA from patients and their parents as previously described^34,35^. The DNA was processed using SureSelect Human All Exon V5 kit or SureSelectXT Human All Exon V5 kit (Agilent Technologies, Santa Clara, CA, USA). Captured DNA (paired-end reads) was sequenced on a HiSeq 2000 or 2500 instrument (Illumina Inc, San Diego, CA, USA). Reads were base called using CASAVA software (Illumina Inc,) and mapped to the human reference genome (GRCh37/hg19) using Novoalign-3.02.04 (for P1 and P2) or MOSAIK (2.2.3)^36^ for P4. Duplicates were marked by Picard 1.92 and variants were called using Genome Analysis Tool Kit GATK (v2.7-4) (P1 and P2) (GATK v.3.2.0)^37^ (P4) following GATK best practice guidelines for DNA sequencing data and annotated using ANNOVAR (version 2014 July 14)^38^. For P4, variants were annotated using Variant Effect Predictor (VEP)^39^ and loaded into GEMINI database (v0.16.0)^40^.

Variants with minor allele frequency of 1% or higher in 1000 Genomes Project (1000 G, 2014 October version), 6500 NHLBI-GO Exome Sequencing Project (EVS) and Exome Aggregation Consortium (ExAC v0.2) were excluded. Only non-synonymous variants, indels and putative splice site variants were considered for further analysis. dbSNP138nonflagged was used to annotate known SNPs. Combined Annotation Dependent Depletion (CADD)^41^ was used to score the pathogenicity of SNVs.

### Segregation and haplotype analyses

The variants in *KIAA0753* were amplified with polymerase chain reaction (primers and PCR conditions available on request) and Sanger sequencing was performed according to standard procedures. Haplotype analysis was analyzed as described earlier^42^.

### Histology and immunohistochemistry of human growth plate

Paraffin-embedded sections of the distal femoral growth plate from P4 and corresponding sections of a human control fetus (the tissues were provided from routine pathology examination of a spontaneous abortion at GA of 19 weeks and 3 days) were obtained. Deparaffinized sections were stained using hematoxylin-eosin according to standard protocols. For immunohistochemical analyses antigen retrieval with near-boiling in citrate buffer for 30 min (for KIAA0753) and pressure-cooking in citrate buffer up to approximately 110 °C (for GAPDH) was performed. After blocking with 3% normal horse serum in PBS and 0.1% Triton-X-100 for 1 h, slides were incubated with primary anti-KIAA0753 antibody (1:100; ab121736; Abcam) and anti-GAPDH antibody (1:100; 14C10; Cell Signaling) overnight at 4 °C, washed with PBS + 0.1% Tween 20, and incubated with secondary antibody (1:400; Cy3-conjugated Donkey anti-Rabbit IgG from Jackson ImmunoResearch Laboratories) for 1 h at room temperature protected from light. After counterstaining with DAPI (1:200) and mounted with Fluoroshield, fluorescent microscopy images were collected using a NikonE600 microscope with Axiovision software (Zeiss) and analysis of images was performed using ImageJ software. As a negative control, the procedure was performed as above but without the primary antibody.

### Generation and maintenance of homozygous knockout *kiaa0753* zebrafish

To analyse the role of Kiaa0753 in skeletal morphogenesis in zebrafish, a mutant line (sa22657) carrying a nonsense mutation in *kiaa0753* was used (European Zebrafish Resource Center, EZRC). Adult zebrafish were maintained on a 14 h day/night cycle at the Karolinska Institute zebrafish core facility. Embryos were produced via light-induced spawning and raised at 28 °C. Fish maintenance was according to standard operating procedures and with permission from the Stockholm Ethical Board for Animal Experiments (protocol number N111/13) all data collection was carried out in accordance with relevant guidelines and regulations.

#### Zebrafish genotyping

DNA collection from adult zebrafish was carried out by clipping a small portion of the caudal fin and extracting total DNA by incubation with proteinase K at a concentration of 0.6 mg/mL in water during 30 minutes at 55 °C, followed by 10 minutes at 80 °C. Embryo DNA extraction was performed by incubation of a portion of embryonic tissue in a solution of 50 mM NaOH during 20 minutes at 55 °C. Sanger sequencing was used to identify the genotype (PCR primers and conditions available on request).

#### Analysis of zebrafish skeletal morphology

Zebrafish larvae were fixed at 5 days post fertilization (dpf) in 4% paraformaldehyde (PFA, Histolab) overnight at 4 °C, rinsed in 1X PBST (phosphate buffered saline), and pigment was removed by incubation with a 3% H_2_O_2_ and 4% KOH solution for 10 minutes. Alcian staining solution (0.1% Alcian blue, 1% HCl in 70% ethanol) was added and left over night followed by one wash in 100% ethanol and embryo rehydration.

Heads were dissected using sharp forceps, mounted in Gelvatol, and imaged using an Olympus IX73 widefield fluorescence microscope. The length of 7 cranial elements was measured using Fiji software. Number of measurements for *sa22657* ^*wt/−*^ was 14 and 11 for *sa22657* ^*mut/mut*^. Statistical analysis was performed using R and t -test.

### *In silico* resources

The URLs for data presented herein are as follows:

Human Gene Mutation Database (HGMD) www.hgmd.org/

Reference Sequence database (RefSeq) www.ncbi.nlm.nih.gov/refseq/

MGI Gene Expression Database www.informatics.jax.org/expression.shtml/

Basic Local Alignment Search Tool (BLAST) http://blast.ncbi.nlm.nih.gov/Blast.cgi/

NHLBI Exome Sequencing Project (ESP) http://evs.gs.washington.edu/EVS/

Exome Aggregation Consortium (ExAC) http://exac.broadinstitute.org/

1000 Genomes Project (1000 G) www.1000genomes.org/

Genome analysis tool kit (GATK) www.broadinstitute.org/gatk/

Annovar http://annovar.openbioinformatics.org/

Gemini, https://gemini.readthedocs.org/en/latest/

ENSEMBL, http://www.ensembl.org/info/docs/tools/vep/index.html

Combined Annotation Dependent Depletion (“CADD”), http://cadd.gs.washington.edu

Genome Aggregation database, http://gnomad.broadinstitute.org

WHO and CDC growth charts, https://www.cdc.gov/growthcharts/who_charts.htm

European Zebrafish Resource Center, http://www.ezrc.kit.edu

ClinVar, https://www.ncbi.nlm.nih.gov/clinvar/

Phenomizer, http://compbio.charite.de/phenomizer/

## Electronic supplementary material

Supplementary information
