# Supplementary material for: Novel KIAA0753 mutations extend the phenotype of skeletal ciliopathies
Source: Sci Rep. 2017 Nov 14;7:15585. doi: 10.1038/s41598-017-15442-1 (PMC5686170; doi:10.1038/s41598-017-15442-1)
Supplement: Supplementary file 1 — Supplementary information [file 41598_2017_15442_MOESM1_ESM.pdf]

**Supplemental information:**

# Novel *KIAA0753* mutations extend the phenotype of skeletal ciliopathies

Hammarsjö A[1,2,¶], Wang Z[3,4,¶], Vaz R[1], Taylan F[1], Sedghi M[5], Girisha KM[6], Chitayat D[7,8], Neethukrishna K[6], Shannon P[9], Godoy R[7], Gowrishankar K[10], Lindstrand A[1,2], Nasiri J[11], Baktashian M[12], Newton PT[13], Guo L[3], Hofmeister W[1], Pettersson M[1,2], Chagin AS[13], Nishimura G[14], Yan L[15], Matsumoto N[16], Nordgren A[1,2], Miyake N[16], Grigelioniene G[1,2,*], Ikegawa S[3,*]

¶ These authors contributed equally to this work

* Corresponding authors, contributed equally to this work

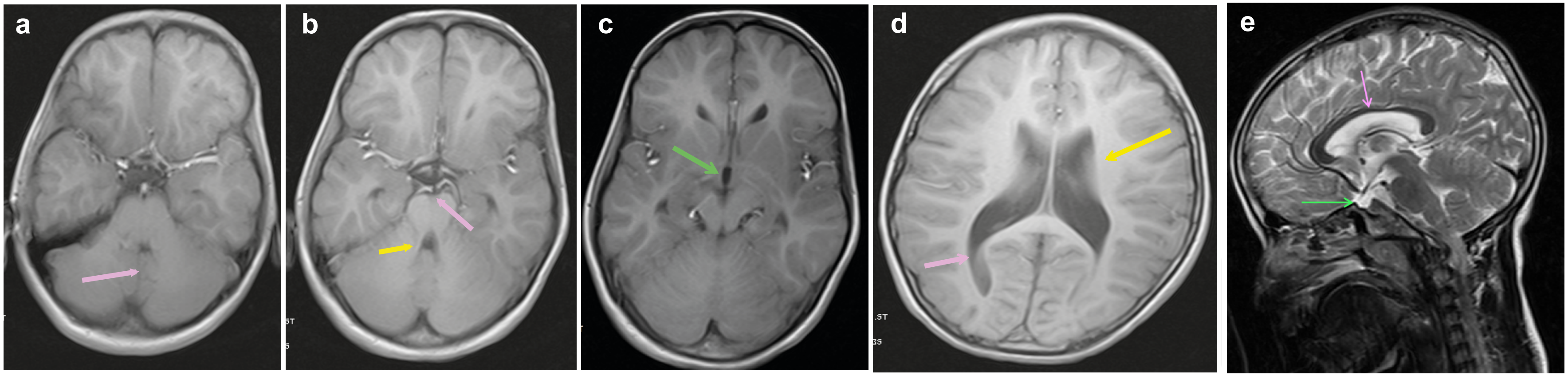


**Supplementary Figure 1. MRI of P1 showing brain abnormalities.**

(a-d) Axial T1-weighted and (e) sagittal T2-weighted images.

(a) Inferior vermian dysplasia and midline cerebellar cleft (pink arrow).

(b) Molar tooth sign, the deepening and enlargement of the interpeduncular fossa (pink arrow) and thickened, maloriented superior cerebellar peduncles (yellow arrow).

(c) Mild dilation of the third ventricle (green arrow).

(d) Elongated occipital horn of the lateral ventricle (pink arrow) and enlarged lateral ventricles (yellow arrow).

(e) Hypoplasia of the pituitary (green arrow) and corpus callosum (pink arrow).

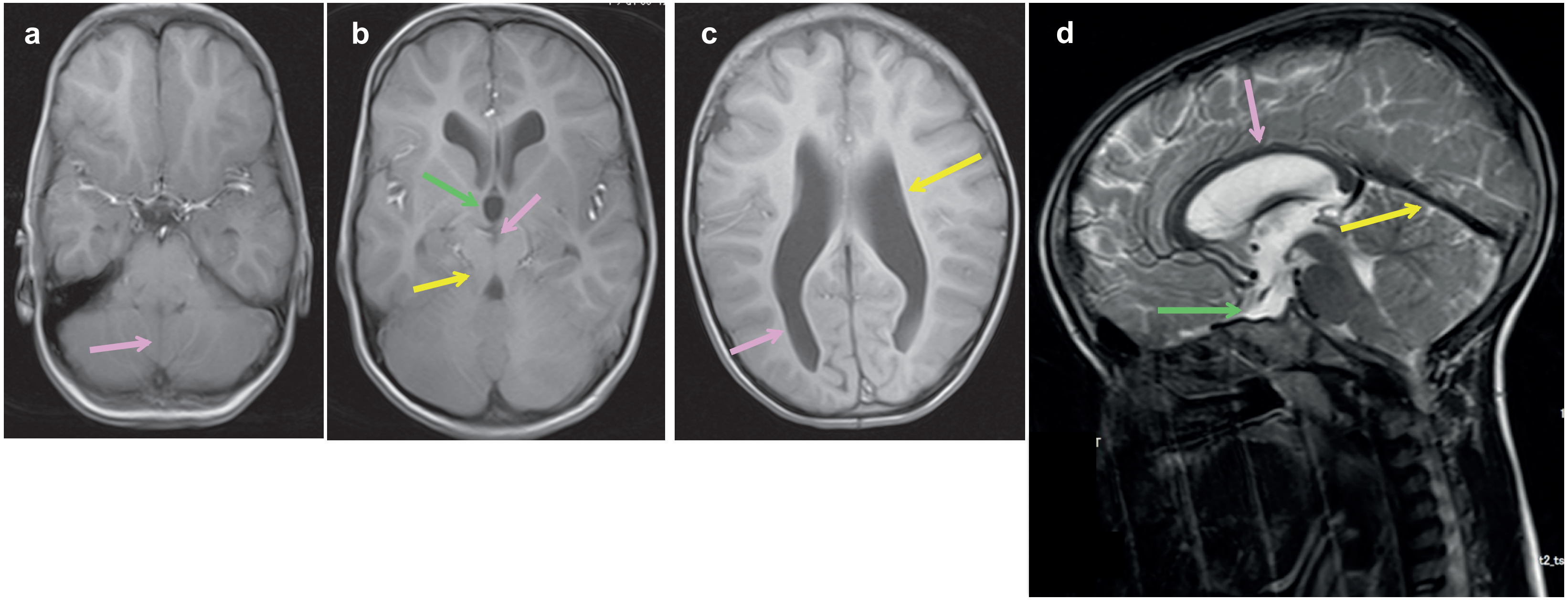


**Supplementary Figure 2. MRI of P2 showing brain abnormalities.**

(a-c) Axial T1-weighted and (d) sagittal T2-weighted images.

(a) Inferior vermian dysplasia and midline cerebellar cleft (pink arrow).

(b) Molar tooth sign, the deepening and enlargement of interpeduncular fossa (pink arrow) and thickened, maloriented superior cerebellar peduncles (yellow arrow) and dilation of the third ventricle (green arrow).

(c) Elongated occipital horn of the lateral ventricles (pink arrow) and enlarged lateral ventricles (yellow arrow).

(d) Hypoplasia of the pituitary (green arrow) and corpus callosum (pink arrow) with upward displacement of the tentorium (yellow arrow).

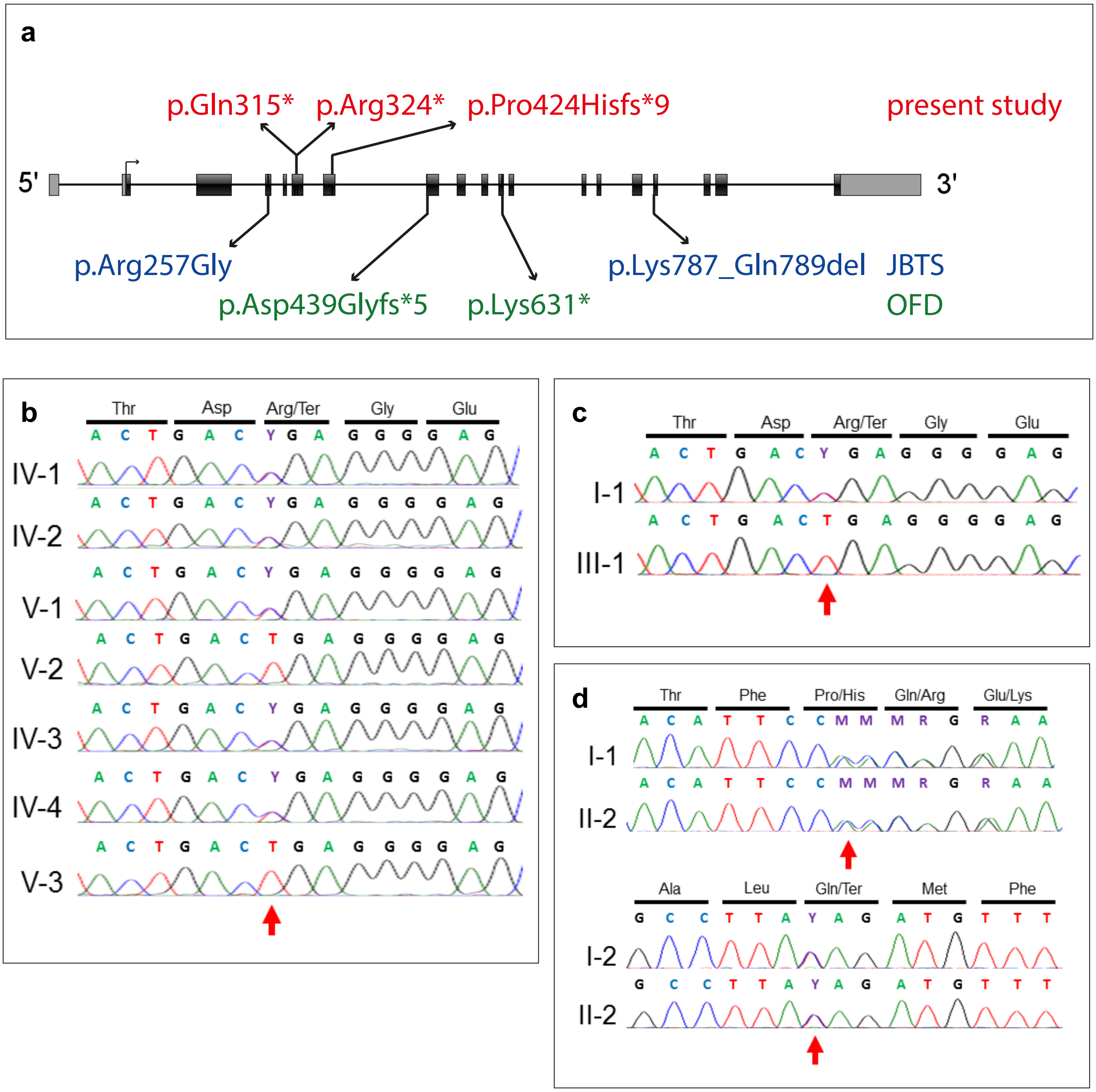


**Supplementary Figure 3. Currently known disease-causing variants in *KIAA0753*.**
(a) Schematic representation of the *KIAA0753* (NM_014894) gene structure and the positions of the currently known disease-causing variants. UTRs are in grey and coding exons (2-19) in black. Variants found in the present study are in red and previously reported variants in Joubert syndrome (JBTS) and orofaciodigital syndrome (OFD) are in blue and green, respectively.
(b) Sanger sequencing of family 1. Parents (IV-1 to IV-4) and one unaffected sibling (V-1) are heterozygous carriers and patients P1 (V-2) and P2 (V-3) are homozygous for variant p.Arg324* (red arrow).
(c) Sanger sequencing of family 2. Father (I-1) was a heterozygous carrier of p.Arg324* and P3 (III-3) was homozygous for the variant (red arrow).
(d) Sanger sequencing of family 3. The father (I-1) was a heterozygous carrier of p.Pro424Hisfs*9 and the mother (I-2) was a heterozygous carrier of p.Gln315*.
P4 (II-2) was compound heterozygous for the variants (red arrow).

**Supplementary Table 1. Summary of present and previously reported patients with mutations in *KIAA0753***

| | Patient 1 | Patient 2 | Patient 3 | Patient 4 | Patient 1 (Chevrier et al. 2016) | Patient 1 (Stephen et al. 2017) | Patient 2 (Stephen et al. 2017) |
|---|---|---|---|---|---|---|---|
| Clinical diagnosis | SKD-JBTS | SKD-JBTS | SKD-JBTS? | SRTD (fetus) | OFD | JBTS | JBTS |
| Gender | Female | Female | Male | Male | Female | Male | Female |
| Birth GA [weeks] | 39 | 39 | Full term | 19+3 (TOP) | 38 | Full term | Full term |
| Length [cm] | 50 (+0.20) | 53 (+1.29) | - | 24.5 | - | 49.5 (-0.25) | 51.5 (+0.75) |
| Weight [kg] | 3.00 (-0.81) | 4.15 (+1.58) | 3.00 (-0.90) | 0.38 | - | 3.87 (+0.63) | 3.52 (+0.21) |
| OFC [cm] | 36 (+0.67) | 39 (+2.33) | - | 19 | - | - | 35.5 (+0.39) |
| Age [years;months] | 1;8 | 2;0 | - | - | - | 7;0 | 1;10 |
| Height [cm] | 68 (-4.19) | 75 (-3.00) | - | - | - | 108.2 (-2.53) | 75.5 (-2.42) |
| Weight [kg] | 8.0 (-3.22) | 9.2 (-2.56) | - | - | - | 18.0 (-1.95) | 9.8 (-1.76) |
| OFC [cm] | 52 (+3.87) | 51 (+2.61) | - | - | - | 52 (+0.00) | 49 (+1.37) |
| Age [years;months] | 5;11 | 5;10 | 6;3 | - | - | 10;6* | 5;0* |
| Height [cm] | 81.0 (-6.72) | 88.0 (-5.18) | 89.0 (-5.36) | - | - | 138.9 (-0.22) | 110.5 (+0.58) |
| Weight [kg] | 12.5 (-3.30) | 14.5 (-2.35) | 13.6 (-3.23) | - | - | 33.8 (+0.05) | 20.7 (+0.76) |
| OFC [cm] | 53 (+1.59) | 52 (+0.92) | 52 (+0.29) | - | - | 56 (+2.13) | 55 (+3.28) |

SKD, skeletal dysplasia; SRTD, short-rib thoracic dysplasia, OFD, orofaciodigital syndrome; JBTS, Joubert syndrome; -, not available; TOP, termination of pregnancy; OFC, occipitofrontal circumference; Z-score in parenthesis after each measurement - calculated according WHO growth standards for infants and children aged 0 to 2 years and CDC Growth Charts: United States for children age 2 years and older). * children responded to growth hormone therapy

**Supplementary Table 2. Radiological findings in present patients**

| | Patient 1 | Patient 2 | Patient 3 | Patient 4 |
|---|---|---|---|---|
| Short ribs/small thorax HP:0000773/HP:0005257 | + | + | + | + |
| Cupped anterior ends of ribs HP:0000907 | + | + | + | + |
| Trident ilia HP:0000946 | + | + | + | NA |
| Spikes at the sacrosciatic notches | + | + | + | NA |
| Narrow sciatic notch HP:0003375 | + | + | + | NA |
| Genu varum HP:0002970 | + | + | + | NA |
| Broad metaphyses HP:0003016 | + | + | + | + |
| Short and bowed tubular bones HP:0003026/HP:0006487 | + | + | + | + |
| Cone-shaped epiphyses HP:0010579 | + | + | + | NA |
| Short metacarpals HP:0010049 | + | + | + | + |
| Platyspondyly HP:0000926 | - | - | - | - |
| NA, not available | | | | |

**Supplementary Table 3. Performance statistics of exome sequencing[a]**

| | Read | | Coverage (%) | | Variations |
|---|---|---|---|---|---|
| Patient | Total (bp) | Mean depth | 10X | 20X | |
| P1 (V:2) | 3132.1 M | 93.6X | 96.1 | 93.1 | NA |
| P2 (V:3) | 3249.0 M | 97.1X | 96.1 | 92.9 | NA |
| P3 (III:1) | 3858.9 M | 85.2X | 96.8 | 91.8 | 38 658 |
| P4 (II:2) | 154.9 M | 252X | 98.4 | NA | 38 904 |
| P4 (I:1) | 146.8 M | 256X | 98.2 | NA | 39 673 |
| P4 (I:2) | 149.0 M | 287X | 98.4 | NA | 38 793 |

[a] Calculated based on the entire coding regions of refGene hg19

**Supplementary Table 4. Haplotype analysis of P1-P3**

| Chr | Position | Ref | Patient1 | Pateint2 | Patient3 | gnomAD allele frequency | dbSNP ID | Location | Gene |
|---|---|---|---|---|---|---|---|---|---|
| 17 | 6330068 | T | T/T | T/T | C/C | 0.7275 | rs2292546 | exonic | AIPL1 |
| 17 | 6331803 | T | T/T | T/T | C/C | 0.6389 | rs8075035 | exonic | AIPL1 |
| 17 | 6364753 | A | G/G | G/G | G/G | 0.8679 | rs11654099 | exonic | PITPNM3 |
| 17 | 6381939 | G | G/G | G/G | A/A | 0.5278 | rs938288 | exonic | PITPNM3 |
| 17 | 6493198 | T | C/C | C/C | C/C | 0.6233 | rs1443417 | exonic | KIAA0753 |
| 17 | 6511781 | A | C/C | C/C | C/C | 0.6136 | rs4796519 | exonic | KIAA0753 |
| 17 | 6513329 | G | G/G | G/G | A/A | 0.3546 | rs2304977 | exonic | KIAA0753 |
| 17 | 6515387 | A | G/G | G/G | G/G | 0.6232 | rs2289642 | exonic | KIAA0753 |
| 17 | 6515454 | C | T/T | T/T | T/T | 0.5582 | rs2289643 | exonic | KIAA0753 |
| 17 | 6524298 | T | T/T | T/T | A/A | 0.3658 | rs9889363 | exonic | KIAA0753 |
| **17** | **6526336** | **G** | **A/A** | **A/A** | **A/A** | **0.00001627** | **rs746068882** | **exonic** | **KIAA0753** |
| 17 | 6531648 | A | G/G | G/G | G/G | 0.6266 | rs2072149 | exonic | KIAA0753 |
| 17 | 6555546 | C | CG/CG | CG/CG | CG/CG | 0.999 | rs56268735 | exonic | C17orf100 |
| **17** | **6607319** | **G** | **A/A** | **A/A** | **G/G** | **0.000008146** | **rs761917087** | **exonic** | **SLC13A5** |
| 17 | 6663895 | C | G/G | G/G | G/G | 0.3938 | rs2271231 | exonic | XAF1 |
| 17 | 6683215 | C | T/T | T/T | C/C | 0.2641 | rs4796555 | exonic | FBXO39 |
| 17 | 6690161 | C | T/T | T/T | C/C | 0.2545 | rs17853331 | exonic | FBXO39 |
| 17 | 6716242 | G | A/A | A/A | G/G | 0.2271 | rs3744395 | exonic | TEKT1 |
| 17 | 6733672 | T | C/C | C/C | T/T | 0.5044 | rs8078571 | exonic | TEKT1 |
| 17 | 6899559 | C | G/G | G/G | C/C | 0.9684 | rs312467 | exonic | ALOX12 |
| 17 | 6902127 | G | G/G | G/G | T/T | 0.0001015 | rs554958955 | exonic | ALOX12 |

Colour code:
Red -> Unshared variants
Blue -> Shared variants
Green -> Pathogenic variant in *KIAA0753*
Yellow -> Pathogenic variant in *SLC13A5* in P1 and P2

Patient 1 and 2 -> Iranian
Patient 3 -> Indian
Shared region = 29.21kb
